# What's So Hot about Recombination Hotspots?

**DOI:** 10.1371/journal.pbio.0020190

**Published:** 2004-06-15

**Authors:** Jody Hey

## Abstract

Recombination is a nearly ubituitous feature of genomes; where and when it occurs can provide insights about its evolution and can affect our ability to identify genes that cause disease

Consider a piece of text, either this one that you are now reading or any other. Surely they are all pretty much alike, in so far as they are all run-on strings of characters. In this same sense, we can envision that all DNA strands are alike because all are monotonous polymers with the same general chemical makeup. Indeed, this is how we think of DNA when considering its basic function of inheritance, in which all parts of all chromosomes must be duplicated and then passed from one cell generation to the next. The capacity for inheritance is fundamentally a consequence of DNA's general molecular structure, and not of its sequence per se, as Watson and Crick (1953), and indeed Muller (1922) long before them, well appreciated. Muller did not know that genes are made of DNA, but he did realize that, whatever genes were made of, they must have a general capacity to replicate, regardless of the information they carry (Muller 1922).

But sequence does matter when DNA fulfills its other, more directly functional role. When the DNA that makes up a gene is exposed and expressed, when a gene is serving its individual function, then the detailed sequence means all.

So where does recombination (Box 1) fit in? Is recombination something that happens to DNA generally? Or does it happen to particular sequences? Bacteria have their chi (χ) sequence, which is a specific series of eight base pairs in the DNA of the bacterial chromosome that stimulate the action of proteins that bring about recombination (Eggleston and West 1997). Similarly, the immunoglobulin genes of mammals have recombination signal sequences that are involved in V-J joining—a kind of somatic recombination involving the joining of a variable gene segment and a joining segment to form an immunoglobulin gene (Krangel 2003). But does normal meiotic recombination depend on the local DNA sequence? In yeast, as well as mammals (mice and humans), the answer is partly yes, for it is clear that chromosomes have local recombination hotspots where crossing over is much more likely to occur than in other places on the chromosome. Recombination hotspots are local regions of chromosomes, on the order of one or two thousand base pairs of DNA (or less—their length is difficult to measure), in which recombination events tend to be concentrated. Often they are flanked by “coldspots,” regions of lower than average frequency of recombination (Lichten and Goldman 1995).

## Diverse Implications of Recombination Hotspots: The Study of Meiosis and the Mapping of Human Disease Alleles

Recombination hotspots are of strong interest to at least two quite different groups of biologists. For geneticists and cell biologists who study meiosis, the existence of recombination hotspots offers a way to learn what other processes are associated with recombination. This is partly how we know that homologous crossovers in yeast and other eukaryotes are initiated by the cleavage of single chromosomes, called “double-strand breaks” (Box 1). It turns out that because of this causal linkage, the hotspots for doublestrand breaks and the hotspots for recombination are one and the same (Game et al. 1989; Sun et al. 1989; Keeney et al. 1997; Lopes et al. 1999; Allers and Lichten 2001; Hunter 2003).

**Figure 1:**
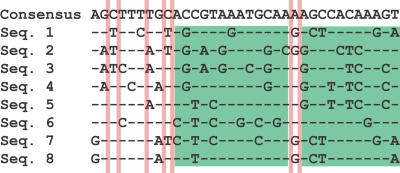
A Hypothetical Example of Eight Aligned Haplotypes for All the SNPs Found in a Region Base positions that are not variable are not shown. Blocks of adjacent SNPs that revealed no evidence of historical recombination are flanked by vertical red bars. Two longer haplotype blocks are indicated in green. The presence of historical recombination was discerned by the four-gamete criterion. In brief, if the haplotypes for two SNPs, each with two bases (e.g., A/G at one SNP and C/T at the second), reveal all four possible combinations (i.e., A-C, A-T, GC, and G-T) then this is evidence that there has been a recombination event between these SNPs in the history of the sample of haplotypes (Hudson and Kaplan 1985). If haplotype blocks are long, then it is possible to represent much of the haplotype diversity using just a small sample of the SNPs found within that region.

For population geneticists, much of the interest in recombination hotspots comes from their possible effect on the patterns of DNA sequence variation along human chromosomes and from the possibility that these patterns could be used to map the position of alleles that cause disease. When multiple copies of the DNA sequence of a gene, or of a larger region of a chromosome, are aligned, they reveal the location and distribution of variation at individual nucleotide positions—single nucleotide polymorphisms (SNPs). Each particular sequence, or haplotype, will carry a configuration for the SNPs for that region (Figure 1). Investigators have long known that SNPs that are adjacent or near each other tend to be highly correlated in their pattern and to exhibit strong linkage disequilibrium (Box 1). It is this linkage disequilibrium that enables scientists to map the locations of mutations that cause heritable genetic diseases. If alleles that cause a disease have the same kind of linkage disequilibrium with nearby SNPs as SNPs generally have with each other, then one could search for genes with disease alleles by looking for a pattern of SNPs that is found only in people who have the disease. This general method for mapping disease alleles is called “association mapping,” and it is basically a search for linkage disequilibrium between disease alleles and other SNPs. Whether or not association mapping works depends on the actual patterns of linkage that occur among SNPs in human populations, and these patterns depend in turn on how much recombination has occurred in the past (as well as on other demographic and mutation processes).

With the advent of larger human haplotype data sets, it has become clear that there are often fairly long regions with very high linkage disequilibrium (Daly et al. 2001; Patil et al. 2001; Gabriel et al. 2002). This pattern of variation has been characterized as occurring in “haplotype blocks,” which are apparent regions of low recombination (or high linkage disequilibrium). Figure 1 shows a hypothetical example of haplotype blocks among eight haplotypes for a series of SNPs found over a region of a chromosome. Given diverse evidence of recombination hotspots in humans, a much discussed question is whether recombination hotspots play a large role in the formation of the pattern of haplotype blocks (Wang et al. 2002; Innan et al. 2003; Phillips et al. 2003; Stumpf and Goldstein 2003). The occurrence of haplotype blocks has inspired the HAPMAP project (http://www.hapmap.org/), which has the goal of identifying a subset of SNPs that capture most of the relevant linkage information in the human genome (IHC 2003). If one had a subset of all common SNPs, with one or two per haplotype block, then this subset would contain much of the available information for association mapping of disease alleles.

## The Evolution of Recombination and (Possibly) Recombination Hotspots

Recombination is a nearly ubiquitous feature of genomes, and a great many theories have been put forward to explain why it would be evolutionarily advantageous for genes to regularly break with one another to join new genes (Barton and Charlesworth 1998). By and large these theories predict that recombination should occur more often where genes occur in higher concentration and that it should happen less often in areas of the genome where genes are spaced far apart. This expectation is roughly born out in the human genome, where recombination rates are higher in regions of the genome with higher gene density (Fullerton et al. 2001; Kong et al. 2002).

To consider the possible evolutionary advantages of individual recombination hotspots, we can draw from theory on the evolution of recombination modifiers. In particular, recent population genetic theory has brought to light some fairly general circumstances for which mutations that raise recombination rates would be favored by natural selection (Barton 1995; Otto and Barton 1997; Otto and Barton 2001; Otto and Lenormand 2002). The basic idea is that linkage disequilibrium can easily occur (for many reasons) between two (or more) polymorphic sites that are under selection. When this occurs, an allele that raises the recombination rate (and decreases the linkage disequilibrium) can cause selection to act more efficiently. If an allele that is under positive or negative selection always occurs with an allele at another locus that is also under selection (i.e., the two loci are in strong linkage disequilibrium), then selection cannot act on one locus independently of the second locus. As new, multilocus configurations of beneficial alleles are generated (by recombination) and increase in frequency by selection, the modifiers of recombination that caused the production of those beneficial configurations increase in frequency with them. A key piece of evidence supporting this kind of theory of the evolution of recombination is directional selection, like that which occurs in artificial selection experiments, which often generates a correlated elevation in recombination rates (Otto and Lenormand 2002).

Connecting these ideas about the evolution of recombination modifiers to the question of recombination hotspots, we come to the possibility that individual hotspots may have arisen as a byproduct of linkage disequilibrium between genes on either side of the hotspot that were under selection. This situation would create a kind of selection pressure favoring recombinant haplotypes and thus also favoring those chromosomes that happen to have a high recombination rate between the selected genes. If true, then we might expect local recombination rates (i.e., hotspots and coldspots for recombination) to fluctuate in location and intensity, in ways that would be hard to precisely predict without knowing what genes have been under selection and what patterns of linkage disequilibria there may have been.

In this light, the paper by Ptak et al. (2004) in this issue of *PLoS Biology* is especially interesting. They report that chimpanzees do not have a recombination hotspot in the TAP2 region where humans have a fairly well characterized recombination hotspot (Jeffreys and Neumann 2002). Ptak et al.'s is a statistical study of linkage disequilibrium in the TAP2 region of chimpanzees and humans, and is less direct than the sperm-typing study of Jeffreys and Neumann (2002). However the contrast in linkage patterns between humans and our closest relatives suggests that recombination hotspots can evolve fairly quickly.

## Functional Constraints on Recombination Hotspots

As appealing as the recombination modifier theory of recombination hotspots may be, there is circumstantial evidence that argues against it and that suggests that recombination hotspots are not directly the byproduct of selection on alleles in linkage disequilibrium. Particularly important in this regard is that some wellstudied organisms (notably the worm Caenorhabditis elegans and the fruitfly Drosophila melanogaster) have not shown evidence of recombination hotspots. If we compare these organisms with yeast and mammals, which do show hotspots, we gain some more insight into the factors affecting the evolution of hotspots.

**Figure 2:**
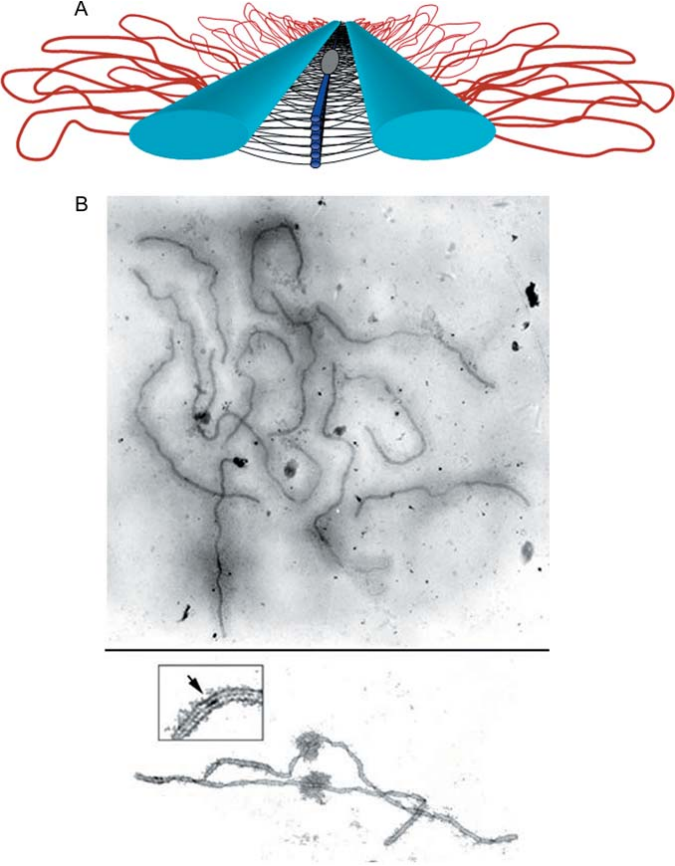
The Synaptonemal Complex (A) Model of the SC. Lateral elements (light blue rods) of homologous chromosomes align and synapse together via a meshwork of transverse filaments (black lines) and longitudinal filaments (dark blue rods). The longitudinal filaments are collectively referred to as the “central element” of the SC. Ellipsoidal structures called recombination nodules (gray ellipsoid) are constructed on the central region of the SC. As their name implies, recombination nodules are believed to be involved in facilitating meiotic recombination (crossing over). The chromatin (red loops) of each homologue is attached to its corresponding lateral element. Because there are two “sister chromatids” in each homologue, two loops are shown extending laterally from each point along a lateral element. (B) Top: Set of tomato SCs. Chromatin “sheaths” are visible around each SC. Bottom: Two tomato SCs. The chromatin has been stripped from the SCs, allowing the details of the SC to be observed. Each SC has a kinetochore (“ball-like” structure) at its centromere. Recombination nodules, ellipsoidal structures found on the central regions of SCs, mark the sites of crossover events (see inset). Images and legend courtesy of Daniel G. Peterson, Mississippi Genome Exploration Laboratory, Mississippi State University, Mississippi State, Mississippi, United States (http://www.msstate.edu/research/mgel/index.htm).

Recall that double-strand breaks are the sites where recombination is initiated during meiosis, and that this is true regardless of the presence of hotspots for both phenomena. Apparently it is the case in yeast and mammals that both recombination and double-strand breaks are also prerequisites for the proper formation of the synaptonemal complex (SC) (Figure 2) and thus for proper orientation of the spindle apparatus and accurate segregation of chromosomes during meiosis (Paques and Haber 1999; Lichten 2001; Hunter 2003; Page and Hawley 2003). In contrast, neither double-strand breaks nor recombination appear to be required for the formation of the SC in D. melanogaster or C. elegans (Zickler and Kleckner 1999; MacQueen et al. 2002; McKim et al. 2002; Hunter 2003; Page and Hawley 2003). Double-strand breaks and recombination do indeed co-occur in these model organisms, and are required for proper chromosome segregation, but they occur after the formation of the SC. Both of these species have broad chromosomal regions where crossing over occurs at higher rates than others, but there have been no reports of local recombination hotspots.

Recombination during meiosis seems to be required for proper chromosome segregation; however, in those organisms where recombination and double-strand-break hotspots occur, these phenomena are also required for proper formation of the SC. It is as if the recombination machinery has been partly co-opted for chromosome alignment in some eukaryotes more so than in others. The implication of these findings is that recombination hotspots are byproducts of other functional constraints associated with the recombination process. This does not rule out the evolutionary theory of recombination modifiers, or that the location and intensity of recombination hotspots may evolve rapidly, but it does suggest that we may not need to invoke the evolutionary modifier theory to explain the existence of recombination hotspots.

## Conclusions

Recombination hotspots co-occur with double-strand-break hotspots in some eukaryotes, and together these phenomena appear to play an important role in the formation of the SC in those organisms. Given the limited phylogenetic occurrence of recombination hotspots (i.e., their occurrence in some, but not all, species), general theories for the evolution of recombination may not be very helpful for understanding the existence of recombination hotspots. However, in those species where they do occur, it is quite possible that recombination hotspots do evolve in location and intensity. Furthermore, the presence of recombination hotspots in humans may have large effects on the length of local patterns of linkage disequilibrium (haplotype blocks) and thus on our ability to map disease alleles by their association with other markers.

##

**Box 1. Glossary**

**Double-strand break:** A break in both strands of a DNA molecule, as distinguished from a break in just one strand.

**Linkage disequilibrium:** A pattern of association between two SNPs or two loci that each have multiple alleles, such that pairs of particular SNPs or alleles, one from each locus, tend to co-occur within individuals or genomes more often than would be expected if the loci are sorting independently of each other.

**Recombination:** The process of one double-stranded DNA molecule joining with another; specifically in the context of meiosis, the process of two homologous chromosomes exchanging large portions of their DNA (this is also called “crossing over”).

## Abbreviations

SC: synaptonemal complex
SNP: single nucleotide polymorphism
